# Association between Hair Cortisol Concentration and Adiposity Measures among Children and Parents from the “Healthy Start” Study

**DOI:** 10.1371/journal.pone.0163639

**Published:** 2016-09-23

**Authors:** Sofus C. Larsen, Jan Fahrenkrug, Nanna J. Olsen, Berit L. Heitmann

**Affiliations:** 1 Research Unit for Dietary Studies at The Parker Institute and Institute of Preventive Medicine, The Capital Region, Copenhagen, Denmark; 2 Department of Clinical Biochemistry, Faculty of Health Sciences, Bispebjerg Hospital, DK-2400, Copenhagen NV, Denmark; 3 The National Institute of Public Health, University of Southern Denmark, Copenhagen, Denmark; 4 The Boden Institute of Obesity, Nutrition, Exercise & Eating Disorders, The University of Sydney, Sydney, Australia; 5 Department of Public Health, University of Copenhagen, Copenhagen, Denmark; McMaster University, CANADA

## Abstract

**Background:**

Previous studies have suggested a direct association between hair cortisol concentration (HCC) and Body Mass Index (BMI), as well as other adiposity measures. However, these studies have mostly been conducted among adult populations.

**Objective:**

To examine the association between HCC and different measures of adiposity among a selected group of children predisposed to obesity and their parents.

**Methods:**

We conducted a cross-sectional study based on 363 children and their parents (301 mothers and 231 fathers) participating in the “Healthy Start” study. Linear regression analysis was used to investigate associations between HCC and adiposity measures while taking into account possible confounding factors. Analyses were performed examining the association between HCC and BMI, fat mass and fat free mass index Z-scores, as well as waist circumference and waist-hip ratio among the children. Likewise, the association between HCC and BMI among the parents was explored. Finally, we examined the association between parental HCC and children’s adiposity measures.

**Results:**

HCC was directly associated with a higher BMI among the fathers (0.49 kg/m^2^ [95% CI: 0.09, 0.90, P = 0.02] per 100 pg/mg) and the mothers (0.93 kg/m^2^ [95% CI: 0.24, 1.61, P = 0.01] per 100 pg/mg). We found no clear evidence of an association between HCC and adiposity measures among children. However, a high maternal HCC was associated with a high fat mass index and low fat free mass index z-score in the offspring (0.14 SD [95% CI: 0.02, 0.26, P = 0.02] and -0.17 SD [95% CI: -0.30, -0.05, P = 0.01] per 100 pg/mg, respectively).

**Conclusions:**

Our study found no evidence of an association between HCC and measures of adiposity among children predisposed to obesity. However, HCC may be directly associated with BMI among men and women, and maternal HCC may be related to a higher fat mass and a lower fat free mass among their children.

## Background

Through the vast majority of human evolution we have lived as hunters and gatherers, and thus natural selection adapted us to a life where physical activity was a necessity for survival and limited food supply was the norm. As a consequence, obesity has been a rare phenomenon for most of human existence, but this began to change with the onset of the industrial revolution, and in 1997 the proportion of obese individuals had reached such an alarming level that the WHO recognized the problem as a global epidemic [1].

Not only the increased availability of food and the generally more sedentary lifestyle has become a reality in a short period of human existence, the type of stress modern humans encounter from work, education and social life are also quite different than those our ancestors faced, while the overall physiological reactions, most likely, have remained unchanged [2].

Following this, a meta-analysis of 14 longitudinal studies found an association between stress and increased measures of adiposity among adults [3]. Likewise, it has been suggested that a higher level of parental stress is associated with childhood obesity, possibly by triggering the child’s own response to stress [4;5]. However, these studies were based on information on stress obtained from questionnaires. Thus, an important limitation to this research area has been the lack of an objective biological measure of chronic stress, making it difficult to disentangle the underlying causes of the associations.

A well-studied pathway in stress response is the Hypothalamic-Pituitary-Adrenocortical (HPA) axis. HPA axis activation starts in the hypothalamus and goes via the pituitary to the adrenal glands, where it leads to increased secretion of cortisol in the bloodstream. Thus, measurement of cortisol in the blood, saliva or urine has hitherto been the preferred biological measure of stress. Although a correlation between salivary cortisol and obesity has been suggested in several studies among children and adults, the direction of this relationship has been inconsistent [6;7]. Also, an important limitation of these measures is that they only provide information of current cortisol level (i.e. an expression of acute stress) [8].

While saliva and plasma cortisol may be indicators of acute stress, hair cortisol concentration (HCC) is a relatively new biomarker for assessing chronic stress, and reflecting exposure during the period of hair growth. Some studies have suggested a direct relationship between HCC and Body Mass Index (BMI: kg/m^2^), waist circumference (WC) and waist-hip ratio (WHR) among adults [9–11], possibly through a direct effect of cortisol on dietary intake [12], influences on metabolism or via psychosocial factors [13]. While the majority of previous studies have been performed in adult populations, a few studies in children have shown similar results. In this regard, a case control study of 20 obese and 20 non-obese 10–12 year old children, found that a higher HCC was observed among the obese than the normal weight and lean children [14]. Moreover, among 85 dyads of children (mean age: 15.4 years [SD: 2.8]) and their primary caregivers, Chen et al. (2015) found direct correlations between caregivers HCC and the BMI or WC of the children [15].

Given this background, the aim of our study was to examine the association between HCC and BMI, fat mass index (FMI: kg/m^2^) and fat free mass index (FFMI: kg/m^2^) Z-scores, as well as WC and WHR among a selected group of children predisposed to obesity. Furthermore, we aimed to examine the association between HCC and BMI among the parents, and the association between parental HCC and adiposity measures among their children.

## Material and Methods

### Study population

This study used data from the Healthy Start project (ClinicalTrials.gov, ID: NCT01583335). The Scientific Ethical Committee of the Capital Region in Denmark decided that according to Section 2.-(1) of the Danish Act on a Bioethics Committee System and the Processing of Bioethics Projects, the project was defined not to be a bioethics project and as a result did not need approval from the Danish Bioethics Committee. Written informed consent to use the collected data for research purpose was obtained from all participants’ parents [16].

A detailed description of the study has been published elsewhere [17]. In brief, the Healthy Start study included children aged 2–6 years at baseline, who were primarily normal weight, but at high risk of future obesity. Based on information from the Danish National Birth Registry and administrative birth forms, children were selected if they either had a high birth weight (> 4000 gr.), an overweight mother (BMI ≥ 28 kg/m^2^), or a mother with low socioeconomic status (≤ 10 years of education). The intervention took place over on average 1.5 year between 2009 and 2011, and consisted of individual guidance in optimizing diet and physical activity habits, reducing chronic stress and stressful events and improving sleep quality and quantity. Information on lifestyle measures was obtained by questionnaires, and HCC was measured on the included children and their parents. The present study was cross-sectional and based on follow-up information from children in the intervention group (n = 320) and the control group (n = 315), and their parents. We had HCC information on a total of 364 children, 335 mothers, and 251 fathers. Information was available on anthropometry and most other variables of interest on a total of 363 children (intervention group [n = 157] control group [n = 206]), 301 mothers and 231 fathers, which were included in the present study.

### Hair cortisol concentration

The concentration of cortisol in hair samples, given as pg/mg hair, was determined by a modification of a previously described protocol [18]. Hair samples were cut from the posterior vertex as close to the scalp as possible. If both parents were not present at the consultation, instructions were given on how to obtain hair samples at home. The samples were stored in aluminum foil, and the scalp end of the hair sample was carefully marked. Between 10 and 20 mg of hair from the 1–2 cm closest to the scalp was accurately weighed and minced finely with scissors. One milliliter of methanol was added and the suspension was incubated overnight at 50°C with a gentle shaking. The following day, the methanol was transferred into a clean tube and evaporated to dryness under nitrogen. The residue was reconstituted in 250 μl PBS buffer (pH 8.0). The cortisol concentration in the resulting buffer solution was determined in duplicate using a commercially available salivary cortisol enzyme-linked immunosorbent assay (ALPCO Diagnostics, Salem, NH, USA). Twenty-seven assays were conducted with an 8.0% intra-assay coefficient of variation. The assay sensitivity was 16.7 pg/mg based on a hair mass of 15 mg. The reproducibility of the assay determined by analysis of aliquots of the same hair samples in different assays was 15%. Hair cortisol concentration was included in the statistical analysis as a continuous variable (units of 100 pg/mg).

### Anthropometry

The children’s heights were measured to the nearest 0.1 cm using a stature meter (Soehnle 5002 or Charter ch200P), and their weights were measured to the nearest 0.1 kg using a mechanical weight or beam-scale type weight (Tanita BWB-800 or SV-SECA 710). The children were measured in under wear only, and were asked to urinate before the weighing. If the child was using diaper, a new diaper was put on before the weighing. Furthermore, WC was measured to the nearest 0.5 cm midway between the lowest rib and the iliac crest. Hip circumference was measured to the nearest 0.5 cm, at the place where the circumference was the largest, seen from the frontal and medial angles. Both WC and hip circumferences were measured in triplicate and a mean was calculated [19]. Bioelectrical impedance was measured on the children at resistance 50 kHz using a SEAC Multiple Frequency Bio impedance Meter (model SFB3 and SFB2 version 1.0), RJL or Animeter (BIA-101 and BIA-103), and skinfolds were measured in triplicate at triceps and subscapular on the left side of the child, using Harpenden Skinfold Caliper or Lange Skinfold Caliper. Procedures have been described in detail elsewhere [17].

From this, we calculated FFM using an equation described by Goran et al. (1996) in young children [20]:

$$FFM\left(kg\right)=\left[0.16\times \left(\frac{H^{2}}{R}\right)\right]+\left(0.67\times weight\right)-\left(0.11\times triceps\right)-\left(0.16\times subscapular\right)+\left(0.43\times sex\right)+2.41\;kg$$

Where H^2^/R is height^2^/resistance in cm^2^/Ω, weight is body weight in kg, triceps and subscapular are skinfold thicknesses in mm, and sex = 0 for girls and 1 for boys. FM was calculated by subtracting FFM from body weight. Finally, the parents reported their own weight and height. In the statistical analyses, we included BMI, BMI Z-score, FMI Z-score, FFMI Z-scores WC, and WHR as continuous variables.

### Potential confounders

We included information on gender and age (continuous variable [*years*]). Furthermore, the parents reported highest level of completed education in 9 categories. Due to very few individuals in some of the groups, we recoded 8 of these categories into 3 levels: *Low education level* (“primary and lower secondary”, “upper secondary”, “one or more short courses”, “skilled worker”), *Medium education level* (“short-term further education [<3 years]” or “medium-term further education [3–4 years]”), and *High education level* (“long-term further education [<4 years]”, “research level”). The 9^th^ category was not recoded, as it included educational information which was not possible to classify according to above (e.g. education completed in foreign countries). We had information on physical activity among children and parents. The parents were asked whether their child liked to be physically active with response options in 5 categories. For this study, we recoded these 5 categories into the following 4 categories: *1) The child never or rarely thinks it is fun being physically active*, *2) the child sometimes thinks it is fun to be physically active*, *3) the child usually thinks it is fun to be physically active*, *and 4) the child always thinks it is fun to be physically active*. Regarding the parents own physical activity level, they were asked how they would describe their level of physical activity in leisure time, over the past year with response options in 4 categories: *1) Regular and vigorous physical activity several times a week*, 2) *some physical activity (cycling to work*, *performing heavy gardening or similar a minimum of 4 hours per week)*, *3) light exercise (e*.*g*. *walking or light gardening a minimum of 4 hours per week)*, *and 4) sedentary activities (e*.*g*. *reading or watching television)*. Finally, we included information on intervention status (*intervention/control*), frequency of hair washing (continuous variable [*weekly frequency*]), and whether participants were using any hair color products (*yes/no*).

### Statistical analyses

BMI z-scores were generated for the children using the Lambda-Mu-Sigma method, which summarizes the changing distributions of BMI by the median, the coefficient of variation and skew expressed as Box-Cox power. Z-scores enable comparison of a measured BMI with adequate gender- and age-specific reference values [19;21]. We applied national reference BMI z-score to the study population. For FMI and FMI z-scores, we calculated standard z-scores as we had no accurate background data for the population z-score distributions.

Gender differences in study characteristics were tested using Wilcoxon rank-sum test for continues variables, and Chi-squared test for categorical variables. Linear regression analyses were used to investigate associations between HCC and BMI among parents and BMI Z-score, FMI Z-score, FFMI Z-score, WC or WHR among the children. All continuous variables were evaluated by model control (investigating linearity of effects on outcome(s), consistency with a normal distribution and variance homogeneity).

First, crude estimates and corresponding 95% confidence intervals (CIs) were calculated (*crude model*), and second, we adjusted for potential confounders (*adjusted model*).

In analyses of the children, the adjusted model included intervention status, gender, age, physical activity and mother's education level. Moreover, possible gender interaction was examined in all analyses by adding product terms to the models, and significant interactions were further examined in stratified analyses.

For the parents, we conducted gender specific analyses, and the adjusted model included intervention status, age, physical activity and education level.

Finally, the same set of analyses was conducted of the association between the parents' HCC and their children's BMI Z-score, FMI Z-score, FFMI Z-score, WC and WHR.

For all sets of analyses, we conducted sensitivity analyses where additional adjustments were made for frequency of hair washing and use of hair coloring products. Furthermore, to maximize the sample size we performed all the crude analyses without excluding participants with missing information on covariates. However, we recognize that this does not allow a direct comparison with the adjusted models. Thus, we performed sensitivity analyses where the crude models were restricted to included participants with complete information on covariates.

All statistical tests were two-sided and P-values <0.05 were considered statistically significant. Analyses were performed using the statistical software package Stata 12 (StataCorp LP, College Station, Texas, USA; www.stata.com).

## Results

Participant characteristics for HCC, anthropometry and covariates for the included children and their parents, stratified by gender are shown in Table 1. There was no significant difference in median HCC (P = 0.08) between boys (95 pg/mg [5–95 percentiles: 26–464]) and girls (89 pg/mg [5–95 percentiles: 23–372]). However, a higher median HCC was found among fathers (150 pg/mg [5–95 percentiles: 56–404]) than mothers (124 pg/mg [5–95 percentiles 47–329]) (p<0.01).

**Table 1:** Study characteristics of chi ldren and parents stratified by gender. Results presented as median and 5–95 percentiles unless stated otherwise.

	Children					Parents				
	Boys		Girls			Fathers		Mothers		
	N	Median (5^th^-95^th^)	n	Median (5^th^-95^th^)	p	N	Median (5^th^-95^th^)	n	Median (5^th^-95^th^)	P
**Age (years)**	200	5 (4, 7)	163	5 (4, 7)	0.50	182	39 (33, 49)	301	38 (31, 45)	<0.01
**Hair cortisol (pg/mg)**	200	95 (26, 464)	163	89 (23, 372)	0.08	231	150 (56, 404)	301	124 (47, 329)	<0.01
**BMI (kg/m2)**	200	16 (14, 18)	163	16 (14, 18)	0.75	231	26 (21, 33)	301	25 (20, 37)	<0.72
**BMI Z-score (SD)**	200	0.3 (-1.0, 1.8)	163	0.4 (-1.0, 1.7)	0.62	-	-	-	-	
**FMI (kg/m2)**	173	3 (1, 5)	144	4 (2, 6)	<0.01	-	-	-	-	
**FMI Z-score (SD)**	173	-0.3 (-1.7, 1.1)	144	0.5 (-1.0, 1.6)	<0.01	-	-	-	-	
**FFMI (kg/m2)**	173	13 (11, 15)	144	12 (10, 15)	<0.01	-	-	-	-	
**FFMI Z-score (SD)**	173	0.1 (-1.2, 1.9)	144	-0.5 (-1.7, 1.5)	<0.01	-	-	-	-	
**Waist circumference (cm)**	188	54 (49, 59)	160	53 (47, 60)	0.01	-	-	-	-	
**Waist-hip ratio**	187	0.91 (0.84, 0.99)	159	0.88 (0.81, 0.96)	<0.01	-	-	-	-	
**PA (% most active)**	177	40%	140	35%	0.81	223	16%	293	11%	0.10
**Education level (% low)**	-	-	-	-		222	32%	294	17%	<0.01

Abbreviations: BMI, body mass index; FMI, fat mass index; FFMI, fat free mass index; PA, physical activity

Difference between boys/men and girls/women was tested using Wilcoxon rank-sum test or Chi-squared test

### Hair cortisol and anthropometry among children

Both the crude and adjusted models showed no statistically significant associations between HCC and BMI Z-score with almost identical association estimates (adjusted β: 0.01 SD [95% CI: -0.04, 0.07, P = 0.70] per 100 pg/mg). Likewise, we found no associations between HCC and FMI Z-score (adjusted β: 0.03 SD [-0.03, 0.08, P = 0.32]), FFMI Z-score (adjusted β: -0.01 SD [-0.07, 0.05, P = 0.69]), WC (adjusted β: 0.10 cm [95% CI: -0.09, 0.30, P = 0.30] per 100 pg/mg) or WHR (adjusted β: -0.001 [95% CI: -0.003, 0.002, P = 0.52] per 100 pg/mg). No evidence of gender interactions was observed in any of the analyses **(**Table 2**)**.

**Table 2:** Association between hair cortisol concentration and anthropometry among children. Results presented in units of 100 pg/mg.

	N	β^1^	95% CI	P	P (sex interaction)
**BMI Z-score (SD)**					
Crude	363	0.01	-0.04, 0.06	0.63	0.72
Adjusted^1^	317	0.01	-0.04, 0.07	0.70	0.45
**FMI Z-score (SD)**					
Crude	317	0.03	-0.03, 0.08	0.39	0.07
Adjusted^1^	280	0.03	-0.03, 0.08	0.32	0.10
**FFMI Z-score (SD)**					
Crude	317	-0.01	-0.07, 0.05	0.83	0.16
Adjusted^1^	280	-0.01	-0.07, 0.05	0.69	0.42
**Waist circumference (cm)**					
Crude	348	0.004	-0.20, 0.21	0.97	0.60
Adjusted^1^	309	0.10	-0.09, 0.30	0.30	0.72
**Waist-hip ratio**					
Crude	346	0.001	-0.002, 0.004	0.39	0.73
Adjusted^1^	308	-0.001	-0.003, 0.002	0.52	0.66

^1^ Calculated using linear regression analyses. Adjusted for intervention status, gender, physical activity, maternal education level and age.

Abbreviations: BMI, body mass index; FMI, fat mass index; FFMI, fat free mass index;

### Parental hair cortisol and BMI

Among both fathers and mothers, their HCC was associated with a higher BMI in both the crude and the adjusted models (adjusted β: 0.49 kg/m^2^ [95% CI: 0.09, 0.90, P = 0.02] and 0.93 kg/m^2^ [0.24, 1.61, P = 0.01] per 100 pg/mg, respectively) **(**Table 3**)**.

**Table 3:** Association between hair cortisol concentration and body mass index among fathers and mothers. Results presented in units of 100 pg/mg.

	N	β^1^	95% CI	P
**Fathers**				
Crude	231	0.67	0.27, 1.07	<0.01
Adjusted^1^	177	0.49	0.09, 0.90	0.02
**Mothers**				
Crude	301	0.73	-0.13, 1.32	0.02
Adjusted^1^	293	0.93	0.24, 1.61	0.01

^1^Calculated using linear regression analyses. Adjusted for interventions status, physical activity, education level and age.

### Parental hair cortisol and child anthropometry

Examination of the association between parental HCC and child anthropometry disclosed no association between fathers HCC and their children´s BMI z-score (adjusted β: -0.07 SD [95% CI: -0.17, 0.04, P = 0.21] per 100 pg/mg), or mothers HCC and children´s BMI Z-score (adjusted β: -0.03 SD [95% CI: -0.15, 0.08, P = 0.59] per 100 pg/mg). Similarly, no associations were observed between paternal HCC and FMI Z-score or FFMI Z-score among their children. However, maternal HCC was associated with a higher FMI Z-score and lower FFMI Z-score (0.14 SD [95% CI: 0.02, 0.26, P = 0.02] and -0.17 SD [95% CI: -0.30, -0.05, P = 0.01], per 100 pg/mg respectively) in the offspring. Moreover, we found no association between fathers HCC and children´s WC (adjusted β: 0.13 cm [95% CI: -0.24, 0.49, P = 0.49] per 100 pg/mg), or mothers HCC and children´s WC (adjusted β: -0.06 cm [95% CI: -0.47, 0.35, P = 0.79] per 100 pg/mg). Finally, we found no association between fathers HCC and children´s WHR (adjusted β: -0.002 [95% CI: -0.007, 0.003, P = 0.35] per 100 pg/mg), or mothers HCC and children´s WHR (adjusted β: -0.002 [95% CI: -0.008, 0.004, P = 0.60] per 100 pg/mg), and no evidence of gender interactions was observed in any of the adjusted analyses **(**Table 4**)**.

**Table 4:** Association between paternal or maternal hair cortisol concentration and anthropometry among their children. Results presented in units of 100 pg/mg.

		Paternal hair cortisol					Maternal hair cortisol			
	n	β	95% CI	P	P -sex interaction	N	β	95% CI	P	P -sex interaction
**BMI Z-score (SD)**										
Crude	251	-0.07	-0.17, 0.04	0.20	0.98	334	-0.05	-0.14, 0.04	0.30	0.002
Adjusted	224	-0.07	-0.17, 0.04	0.21	0.98	297	-0.03	-0.15, 0.08	0.59	0.30
**FMI Z-score (SD)**										
Crude	218	0.01	-0.11, 0.12	0.90	0.51	291	0.16	0.04, 0.29	0.01	0.10
Adjusted^1^	200	0.04	-0.07, 0.14	0.49	0.70	262	0.14	0.02, 0.26	0.02	0.73
**FFMI Z-score (SD)**										
Crude	218	-0.05	-0.17, 0.06	0.36	0.90	291	-0.17	-0.30, -0.04	0.01	0.52
Adjusted^1^	200	-0.07	-0.18, 0.05	0.26	0.70	262	-0.17	-0.30, -0.05	0.01	0.69
**Waist circumference (cm)**										
Crude	236	0.04	-0.36, 0.43	0.86	0.65	320	-0.19	-0.64, 0.25	0.39	0.40
Adjusted	217	0.13	-0.24, 0.49	0.49	0.72	290	-0.06	-0.47, 0.35	0.79	0.59
**Waist-hip ratio**										
Crude	235	-0.004	-0.009, 0.002	0.18	0.37	318	0.000	-0.006, 0.006	0.94	0.60
Adjusted	216	-0.002	-0.007, 0.003	0.35	0.44	313	-0.002	-0.008, 0.004	0.60	0.06

^1^ Calculated using linear regression analyses. Adjusted for intervention status, gender, physical activity, maternal education level and age

Abbreviations: BMI, body mass index; FMI, fat mass index; FFMI, fat free mass index;

Further adjustment for use of hair coloring products and frequency of hair washing had no influence on the statistical significance of the observed associations. Likewise, sensitivity analyses, where the crude models were restricted to included participants with complete information on covariates, did not change the observed associations (results not shown).

## Discussion

There is growing evidence that determination of HCC is an objective tool to determine systemic cortisol exposure over preceding months, and that it could be a biomarker for prolonged stress [18;22;23]. We examined the associations between HCC and different measures of adiposity. In a population of 363 children predisposed to obesity and their parents, we found no clear evidence of an association between HCC and adiposity measures among children. However, HCC was directly associated with BMI among both the fathers and the mothers. Moreover, our data suggests that maternal HCC is related to a higher fat mass and a lower fat free mass among their children.

Results from studies investigating associations between HCC and measures of adiposity among children aged 2–6 years have not previously been reported. However, in a case control study of 20 obese and 20 non-obese 10–12 year old children, a higher HCC was observed among the obese children [14]. In our larger sample of children, we were unable to confirm this finding. However, the significant finding of an association between HCC and BMI among the fathers and mothers is consistent with results from previous studies suggesting a positive association between HCC and BMI, WC and WHR among adults [9–11]. Hence, our study provides further evidence for a link between elevated HCC and obesity among adults. Our population mainly consists of children with a BMI in the healthy range, while the population of parents was enriched in obese subjects. This may be explained by the design of the Healthy Start study, where maternal pre-pregnancy overweight was one of the inclusion criteria. Moreover, as shown in Table 1 the children had a relatively narrow range in BMI compared to their parents. Thus, it is possible that an association between cortisol and BMI is only present among children who are already overweight. Lastly, we found a lower median HCC as well as a larger range in data among the children than their parents. In contrast to our results, a previous study found higher HCC values among children than adults [24]. The lower median HCC among children than parents in our data could be due to the greater proportion of overweight parents than children. Moreover, consistent with our findings there data also revealed a larger SD among children than adults [24]. We found no literature clarifying the biological cause of this, nor have we found studies comparing the validity of the HCC measure among young children with adults. One might speculate that various factors such as greater differences in hair texture and hair growth among children could reduce the precision of the measure.

How HCC is linked to adiposity remains to be clarified. Personality characteristics, coping skills, education and social class are involved in both development of stress and obesity, and such factors may be the underlying cause of the association [25]. Moreover, a possible explanation for the relationship is that hyper-activation of the HPA-axis affects body weight through increases in food intake. Infusion of corticotropin-releasing hormone stimulates cortisol release and subsequent food intake, and it has been suggested that cortisol itself may directly affect dietary intake [12]. Additionally, it is well known that obesity is often followed by chronic inflammation [26;27]. There is some evidence of a crosstalk between the HPA axis and inflammatory response, suggesting that pro-inflammatory cytokines can stimulate the HPA axis, while cortisol decreases the production of cytokines and other inflammatory mediators [28]. Thus, the relationship between HCC and adiposity measures may be mediated by the inflammatory response [13]. Previous studies have suggested that early in life, parental stress may mediate childhood overweight and obesity [4;5;29]. Likewise, as stated above, Chen et al. (2015) found direct correlations between caregiver HCC and their children’s body weight, BMI and WC. In our study, HCC of the parents and BMI z-score, WC or WHR of the children were not associated [15]. However, our results suggest a link between maternal HCC and a higher fat mass as well as a lower fat free mass among their children. Thus, further studies of intergenerational associations as well as the possible underling mechanisms are needed.

The present study has several strengths, including data on HCC measures from a large number of children and their parents, information on several adiposity measures in addition to questionnaire data on sociodemographic and lifestyle factors, allowing us to adjust for potential confounding. However, our study also has some limitations. In this regard, although the HCC measurements reflect stress level in the months prior to the BMI measures, our analyses were essentially cross-sectional making it impossible to assess causality. Although this study included information on a relatively large sample of children and their parents, it is possible that we overlooked some associations because of a lack of statistical power. Nevertheless, the generally quite narrow CIs suggest that it is less likely that we overlooked any noteworthy or clinically relevant associations. In addition, as in other observational studies, we cannot rule out that unmeasured or residual confounding may have affected our results. In this regard, we had no information available on parental alcohol or smoking habits, and we cannot rule out confounding from these factors. Moreover, it is not possible to get an exact measure of physical activity using questionnaire data. Thus, we cannot reject that residual confounding from physical activity has affected our results. This could particularly be the case in analyses of the children, where we adjusted for the parents' perceptions of whether their child liked to be physically active, which is not necessarily a precise measure of the actual amount of physical activity the child performed. However, one study in children from the UK showed that finding physical activity enjoyable was a motivator for active playing [30], and a review from 2012 found that motivation correlates with physical activity [31]. Nevertheless, as we generally found quite similar estimates in the crude and adjusted analyses, we would not expect residual confounding to be a major issue in this study. Likewise, some measurement error may also be present for the body composition measure. Bioelectrical impedance is not a gold standard for the measurement of body composition. However, inaccuracy in our outcome measure cannot explain the observed association between maternal HCC and their children’s body composition, since such measurement errors will not bias the estimate, but only lead to wider CIs (i.e. reducing the likelihood of significant findings). Finally, our analyses were conducted in a selective population of children predisposed to obesity, and results may not be generalizable to other groups.

In conclusion, we found no evidence of an association between HCC and adiposity measures among children predisposed to obesity. However, HCC was directly associated with BMI among fathers and mothers, and maternal HCC was related to a higher fat mass and a lower fat free mass among their children. Further research is needed to determine the causality and underlying mechanisms of the relationships between HCC and adiposity measures.
